# Relevance of Epstein-Barr Virus (EBV) miRNAs in EBV-Infected B Cells and B-Cell Lymphomas

**DOI:** 10.3390/cancers18060962

**Published:** 2026-03-16

**Authors:** Nohora Juliana Rueda-Forero, Joost Kluiver, Marije Koning, Anke van den Berg, Arjan Diepstra

**Affiliations:** 1 Department of Pathology and Medical Biology, University of Groningen, University Medical Center Groningen, 9713 GZ Groningen, The Netherlands; n.j.rueda.forero@umcg.nl (N.J.R.-F.); m.koning-3@umcutrecht.nl (M.K.); a.van.den.berg01@umcg.nl (A.v.d.B.); a.diepstra@umcg.nl (A.D.); 2 Facultad de Ciencias Médicas y de la Salud, Instituto de Investigación MASIRA, Universidad de Santander, Bucaramanga 680003, Colombia; 3 Lymphoma Research Groningen (LRG), University of Groningen, University Medical Center Groningen, 9713 GZ Groningen, The Netherlands

**Keywords:** Epstein–Barr virus, microRNAs, B cells, B-cell lymphoma

## Abstract

Viral infection is a critical early event in Epstein–Barr virus (EBV)-positive B-cell lymphomas. While latent EBV proteins are known to promote cancer development, the role of EBV-encoded microRNAs (miRNAs) is not yet clear. These miRNAs are reported to regulate viral persistence, immune evasion, B-cell survival, and growth. This review compiles evidence on the role of EBV miRNAs in B cells and B-cell lymphomas, including their known target genes, and their effects on cancer-related pathways. By combining profiling studies and results from laboratory models, we highlight how EBV miRNAs might contribute to lymphoma development. Overall, this review provides a comprehensive overview of the biology of EBV-positive B-cell lymphoma, and current knowledge supports a critical role for EBV miRNAs in B cell transformation.

## Simple Summary

Epstein–Barr virus (EBV) is a common virus that infects specific types of white blood cells in a high percentage of the population. In some cases, EBV-infected B cells can transform and develop into specific types of B-cell lymphoma. Researchers have discovered that EBV produces small RNA molecules called microRNAs, which help the virus evade recognition by the immune system, regulate infection patterns, and induce persistent growth of the infected host B cell. Together, these processes promote the development of B-cell lymphoma. In this review, we summarize current knowledge of the roles of these microRNAs in infected B cells and their contributions to lymphoma development.

## 1. Introduction

The Epstein–Barr virus (EBV), also known as human herpesvirus 4, is a double-stranded DNA virus that belongs to the gamma herpesvirus subfamily. EBV can infect epithelial cells and has a preference for human naive B cells. When naive B cells are infected, they differentiate into memory B cells, leading to the virus’s persistence in the host’s body throughout their lifetime. After the initial infection, EBV establishes a latent infection in about 90% of the human population [1]. In vivo, the number of infected B cells is low due to effective targeting by EBV-specific T cells [2]. The virus persists in about 1 out of 10,000 B cells. EBV plays a critical role in various malignancies, including B-cell lymphoma and nasopharyngeal cancer. The association of EBV with specific cancer types varies in different geographic areas [3].

EBV infection is initiated upon binding of the viral gp350/220 protein to CD21, which is expressed on the membrane of target cells. This is followed by binding of the gp85, gp25, and gp42 viral proteins to class II human leukocyte antigen (HLA-II). This process triggers the fusion of the viral envelope with the cell membrane, allowing the virus to enter its target cell. The viral genome circularizes into episomes and resides in the nucleus. EBV first enters a lytic phase characterized by the production of virions, which is followed by a transition to a quiescent latent state featuring limited expression of viral genes [1]. During the latent state, replication of the EBV genome occurs simultaneously with division of the infected B cell [4]. Latent EBV proteins promote proliferation and thereby facilitate the expansion of infected B cells. Latent infection is the default program in memory B-cells and seems sufficient for persistence of EBV [5].

## 2. EBV Infection and EBV-Driven Lymphomagenesis

EBV infection can be considered an early event in the development of EBV-positive B-cell lymphoma. Latently infected naïve B cells have a significantly higher likelihood of developing into B-cell lymphoma than uninfected B cells. The in vivo transforming capability of EBV is strongly related to the germinal center reaction. Normal B cells undergo several stages of differentiation and selectionin order to mature from a naïve B cell to a plasma or memory cell. The germinal center reaction is crucial in this process. Germinal center B cells are characterized by somatic hypermutation and class-switch recombination of the immunoglobulin gene loci. Although tightly regulated, these processes can sometimes lead to off-target mutations in lymphoma driver genes [6]. EBV drives naïve infected B cells into the germinal center and induces extended survival of germinal center B cells, potentially facilitating the accumulation of genetic aberrations. Consistent with this increased risk of genomic instability, most EBV-associated lymphomas originate from germinal center B cells.

To date, significant gaps remain in understanding the molecular mechanisms of EBV-induced B-cell transformation. EBV-encoded proteins have been shown to play important roles in this process. Although Latent Membrane Protein 1 (LMP1) and Epstein–Barr Nuclear Antigen 2 (EBNA2) proteins can transform B cells [7], cells with ectopic expression of only these two genes do not proliferate to the same extent as cells infected with EBV virions. This suggests that additional factors contribute to the transformation of B cells. Over the past two decades, it has become clear that EBV-encoded miRNAs might also play a role in EBV-driven lymphomagenesis [8].

## 3. EBV Genomic Structure and miRNAs

**Figure 1:**
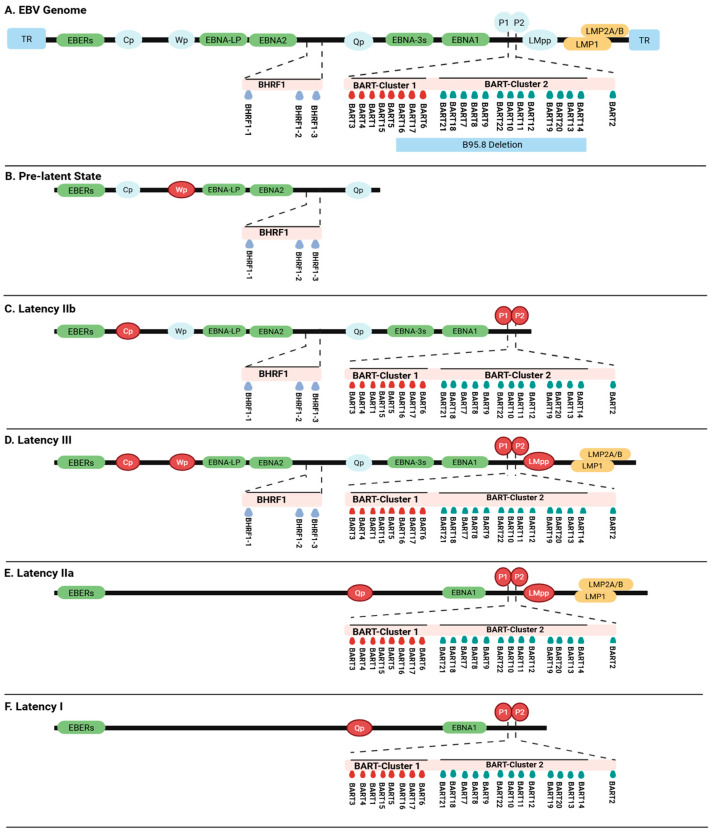
Schematic overview of the genomic structure of the latency type-associated Epstein–Barr virus (EBV) genes and expression programs per latency type. The EBV genome is presented in a linear manner, with the terminal repeats (TRs) at its end. Light blue ovals indicate promoters, while coded regions are presented as colored boxes (green and yellow). The BamHI Fragment H Rightward Open Reading Frame 1 (BHRF1) and BamHI-A Rightward Transcript (BART) microRNA (miRNA) clusters are expanded below the genomic region. The figure contains horizontal panels labeled (**A**–**F**), representing: (**A**) EBV Genome: A linear overview of the entire Epstein–Barr virus genome, depicting major latent and lytic gene clusters, promoters, and microRNA regions. (**B**) Pre-latent State: Illustrates promoter usage and gene expression in newly infected B cells before full establishment of latency. (**C**) Latency IIb: Shows gene and promoter activity typical of the latency IIb stage, with characteristic expression patterns. (**D**) Latency III: Displays the most transcriptionally active latency program, featuring expression of all EBV Nuclear Antigens (EBNAs), Latent Membrane Proteins (LMPs), and both BHRF1 and BART microRNA clusters. (**E**) Latency IIa: Depicts promoter activity and viral gene expression for the intermediate latency IIa state, with restricted EBNA and LMP usage. (**F**) Latency I: Highlights the most restricted latent program, with minimal gene expression (typically only EBNA1 and select noncoding RNAs such as EBV-encoded small RNAs (EBERs) and BART microRNAs). Each panel visually differentiates gene promoter activity (red for active) and expansion of microRNA clusters beneath the corresponding regions, summarizing expression programs characteristic of each latency type in B cells. The EBV promoters referenced are: Qp (EBNA1 Q promoter), P1/P2 (LMP1 promoters 1 and 2), LMpp (LMP promoter), Cp (EBNA C promoter), and Wp (EBNA W promoter). The figure was created using BioRender.com.

The EBV genome is 170–180 kb and encodes approximately 100 viral proteins [9]. Figure 1A offers a schematic overview of the genomic structure related to the latency type-specific expression patterns of EBV. EBV-encoded small RNAs (EBERs) contain intragenic transcriptional control regions for RNA polymerase III and are transcribed during all latency stages [5]. The W promoter drives an initial burst expression of Epstein–Barr nuclear antigen (EBNA), particularly EBNA2 and EBNA-leader protein (Figure 1B) [1]. The transition to the latency IIb program begins with the C promoter, which drives expression of EBNA1, 2, 3A, 3B, 3C, and EBNA-leader protein (Figure 1C). Following this transition, EBNA2 activates the latent membrane protein promoters, leading to the expression of latent membrane protein 1 (LMP1) and LMP2A/B, resulting in a latency III program (Figure 1D) [10]. Latency IIa-infected cells express EBNA1, LMP1, and LMP2A/B. The expression of EBNA1 in this latency type is driven by the Q promoter (Figure 1E) [11]. Silencing of the latent membrane protein promoters is facilitated by epigenetic mechanisms, such as DNA methylation, which limit viral gene expression to EBNA1 exclusively. This allows progression to latency type I (Figure 1F) [12].

Besides encoding multiple protein-coding genes and EBERs, the EBV genome also encodes 25 miRNA genes. MicroRNAs (miRNAs) are small non-coding RNAs (~22 nucleotides) that post-transcriptionally regulate gene expression by binding to target mRNAs. Primary miRNA host transcripts were processed to mature miRNAs in two enzymatic steps by Drosha and Dicer [13]. The first step, from primary miRNA to precursor miRNA, is dependent on a stem-loop-like structure. The second step, which is cleavage of the loop region, results in the final mature miRNA, which is incorporated into RNA-induced silencing complexes (RISC). miRNAs generally bind to the 3′-untranslated regions of their target genes through partial homology [13]. The degree of homology is especially high for nucleotides 2 to 8 of the miRNAs, which represent the so-called seed region. The miRNA–target gene interaction results in the degradation of the mRNA or translational repression, in both cases leading to decreased expression of the target protein. miRNAs have been implicated in virtually all homeostatic processes in human cells [14], while dysregulation of miRNAs has been widely implicated in disease [15].

Unlike many human miRNAs, both strands of most EBV miRNA precursors (guide and passenger) are considered functional [16]. The 25 precursors result in a total of 44 mature miRNAs. EBV miRNAs are clustered in two regions of the EBV genome, and their expression is associated with different latency stages of the virus. The BamHI fragment H rightward open reading frame 1 (BHRF1) encodes three miRNA precursors (BHRF1-1 to -3) that generate four mature miRNAs. The BamHI fragment A rightward open reading frame (BART) contains 22 miRNA precursors (BART1 to 22) that produce 40 mature miRNAs. The BART region can be further subdivided into two subclusters (BART cluster 1 and 2) and one separate precursor (BART2) [17]. Expression of BART miRNAs is regulated by two TATA-less promoter regions (P1 and P2), which are active throughout all latency states (Figure 1) [17,18]. Expression of BART miRNAs is ubiquitous in latency types I, II, and III. The W promoter regulates the expression of the BHRF1 miRNA cluster and is specific for latency type III [10,19,20].

Interestingly, some EBV miRNAs share sequence homology with human miRNAs, resulting in an overlap in their target gene repertoire (Table S1). Seed sequences of 14 EBV miRNAs are similar or identical to seed sequences of human miRNAs. For example, miR-BART5-5p exhibits seed homology with hsa-miR-18a/b-5p [21], miR-BART1-3p shares its seed with hsa-miR-29a/b/c-5p, and the seed of miR-BART22-3p is identical to the seed of hsa-miR-520d-5p and hsa-miR-524-5p [22,23].

Genome-wide experimental approaches, combined with in silico predictions such as High-Throughput Sequencing of RNA isolated by Crosslinking Immunoprecipitation (HITS-CLIP) experiments, have demonstrated that EBV miRNAs can target both EBV and human genes. Interestingly, EBV and human miRNAs showed a substantial overlap in their target gene repertoires. For instance, there is a 50% overlap between the human genes targeted by EBV miRNAs and those targeted by the oncogenic miR-17-92 cluster. EBV-derived miRNAs, which make up 25% of all miRNAs in latency III B cells, collectively target only three EBV latency transcripts. Interestingly, these EBV latency transcripts can also be targeted by host cell miRNAs [24]. High-throughput studies have expanded the catalog of validated EBV miRNA targets [25,26,27]. Curated databases such as VIRmiRNA [28], which focuses specifically on viral miRNA targets, and TarBase (https://dianalab.e-ce.uth.gr/tarbasev9, accessed on 5 March 2026), provide comprehensive overviews of currently known interactions [29].

## 4. Models to Study the Role of EBV Genes

Early events in EBV-driven B-cell lymphoma development can be modeled using lymphoblastoid cell lines (LCLs). These arise spontaneously from cultured EBV-infected B cells or are generated through deliberate in vitro infection of B cells [30].

While LCLs exhibit high proliferation rates in vitro and serve as valuable tools for studying EBV biology in B cells, they do not precisely reflect EBV-positive lymphomas. Unlike the restricted latency patterns typical of EBV-positive lymphomas, LCLs display a fixed latency III program with a broad expression of viral genes. Most importantly, LCLs lack the accumulation of genetic mutations in genes typically observed in B-cell lymphomas. Furthermore, LCLs do not interact with the tumor microenvironment, which is critical for the behavior and progression of lymphoma cells [31].

In vitro models, including engineered cell lines and genetically modified EBV strains, offer essential platforms for examining the roles of specific viral genes. Patient-derived EBV strains have been extensively used as model systems to study the role of specific EBV genes. The Mutu strain has been used to investigate stage-specific gene functions in latency type switching [32]. Other commonly used viral strains, such as B95.8 and M81, have also been instrumental in elucidating the role of EBV miRNAs. The B95.8 strain has a ~12 kb deletion covering the full loss of BART cluster 2 and a partial loss of BART cluster 1, eliminating 16 BART precursor miRNAs (see Figure 1A) [33]. B95.8 cells induce a predominant latent infection pattern in B cells in vitro, and this strain is frequently used to generate LCL cell lines. In vitro cultured primary EBV-infected B cells show genomic diversity converging toward B95.8-like genomes within weeks, particularly in latency genes [33]. In contrast, clinical isolates frequently retain intact BART clusters with geographic variation [34]. The M81, a naturally occurring EBV isolate from a Chinese nasopharyngeal carcinoma patient, exhibits spontaneous lytic replication in B cells (40-fold higher infectious virus) and enhanced epithelial tropism, which is driven by specific polymorphisms and efficient translation of BALF5 [35,36]. M81 has intact BART miRNA clusters and has a ~5-fold lower expression of the BHRF1 miRNAs. This strain has been used to model clinically relevant lytic phenotypes [35,36]. In addition, genetically engineered EBV mutants have enabled functional studies of specific genes, such as the miR-BHRF1 cluster knock-out EBV strain (EBV-Δ123) [37]. Studies using in vitro EBV models have indicated that EBV miRNAs contribute to interconnected functional axes: regulating EBV life cycle, evading immune detection, and controlling cellular growth and survival. Comprehensive overviews of EBV-encoded miRNA–target interactions across various pathologies have been reviewed elsewhere [38,39]. In this review, we specifically focused on proven miRNA-mRNA interactions critical for B-cell functioning and EBV-positive lymphomas (Table 1). Below, we discuss specific interactions related to the EBV life cycle, modulation of the immune response, and regulation of growth and apoptosis of B cells.

### 4.1. EBV miRNAs Regulating the EBV Life Cycle

Several studies have demonstrated a crucial role of EBV miRNAs in the life cycle of EBV, by regulating replication and the transition between lytic and latent infection patterns. One of the EBV miRNAs implicated in this process is miR-BART2 [40]. The target gene responsible for the observed phenotype is BALF5, which is the viral DNA polymerase critical for viral replication. Overexpression of miR-BART2 during the lytic cycle resulted in a strong reduction in BALF5 expression and a 20% reduction in the production of viral particles [40]. Bioinformatics-based analysis focusing on BART miRNAs indicated potential targeting of BZLF1, the key protein for lytic replication, and BRLF, a transcriptional activator of lytic genes, by 12 EBV BART miRNAs [41]. Further experimental validation indicated that only miR-BART20-5p effectively targeted both transcripts and regulated progeny virus production [41]. Another study supported these findings using the M81 EBV strain. BART miRNAs were shown to be downregulated in replicating cells, and an increase in the proportion of spontaneously replicating cells was observed in cells infected with BART miRNA knockout virus compared to the wild-type virus [35]. Although both BART clusters reduced the expression of BZLF1, the effect was more prominent for BART cluster 1 than for BART cluster 2 [35]. In another study, miR-BART6-5p was shown to play a critical role in establishing and maintaining persistent EBV infection by targeting Dicer [42]. This finding was unexpected since Dicer is a key enzyme in processing nearly all pre-miRNAs into mature miRNAs, including those from the host cell. The authors proposed a feedback loop in which miR-BART-6-5p induced suppression of global miRNA production, leading to reduced levels of miR-BART-6. Inhibition of miR-BART-6-5p led to a transition from latency types I and II to the more immunogenic latency type III or to lytic infection [42].

The BHRF1 microRNA cluster promoted early survival and transformation of EBV-infected B cells by regulating BHRF1 protein levels and modulating EBNA-LP expression. In wild-type EBV-infected cells, Drosha processing of pri-miR-BHRF1-2 and -3 cleaves the primary BHRF1 transcript, resulting in low BHRF1 mRNA and protein levels early after infection [43]. A similar effect on BHRF1 protein was observed upon deletion of only the pri-miR-BHRF1-2 region [44]. Drosha-mediated cleavage of the pri-miR-BHRF1 cluster also destabilized EBNA-LP mRNA and protein. This shared mechanism is related to the genomic structure of BHRF1 and EBNA-LP, which are alternatively spliced transcripts from the same locus that also include the BHRF1-3 miRNAs. Thus, both proteins are regulated by Drosha processing of the BHRF1-3 miRNAs in cis [44,45].

Together, these findings show that EBV-encoded miRNAs regulate viral replication, the balance between latent and lytic infection, and viral gene expression programs that contribute to the survival of EBV-infected B cells. By modulating both viral and host genes, these miRNAs help maintain persistent infection and influence viral latency dynamics.

### 4.2. EBV miRNAs Modulate EBV-Directed Immune Responses

Several EBV miRNAs have been shown to regulate anti-EBV immune responses by targeting human genes and thereby affecting CD4 and CD8 T cell responses. For example, miR-BART1, miR-BART2, and miR-BHRF1-2 directly repress IL-12B, a proinflammatory cytokine critical for the differentiation of naive CD4 T cells into Th1 cells [46]. In line with this finding, repression of Th1 differentiation was observed in a co-culture of naïve CD4 T cells with B95.8-infected B cells compared to B cells infected with a modified B95.8 strain in which the deleted microRNAs were reintroduced. This attenuated anti-EBV cytotoxic responses mediated by CD8 T cells, which rely on help from Th1 cells [46]. Direct regulation of IFI30, LGMN, and CTSB by miR-BART1, miR-BART2, and miR-BHRF1-2 resulted in a decrease in antigen presentation by HLA class II. This was caused by interference with lysosomal protein degradation and downregulation of HLA class II and co-stimulatory molecules. As a result, activation of CD4 T cells was reduced [46]. Next to their effects on CD4 T cells, EBV miRNAs also protect EBV-infected B cells from CD8 T cell-mediated killing. B cells infected with the B95.8 strain consistently exhibited improved survival when cocultured with autologous CD8 T cells compared to B cells infected with an EBV strain lacking miRNAs [47]. This is thought to occur due to the direct targeting of the intracellular transporter of antigenic peptides, TAP2, by miR-BHRF1-3 and miR-BART-17, as well as a more general indirect downregulation of the entire TAP complex by EBV miRNAs [47]. MiR-BHRF1-2-5p inhibited IL-1 signaling by directly targeting the IL-1 receptor 1 [48]. This was postulated to dampen inflammatory responses and contribute to immune evasion. Additionally, it has been shown that miR-BHRF1-2-5p can regulate the expression of the inhibitory checkpoint ligands PD-L1 and PD-L2 [49]. While LMP1 is known to induce PD-L1, it is thought that miR-BHRF1-2-5p may fine-tune the expression of the ligands during specific differentiation stages of EBV-infected B cells [49]. Thus, multiple EBV miRNAs are involved in modulating and decreasing antiviral immune responses.

### 4.3. EBV miRNAs Regulate Growth and Apoptosis by Targeting Host Transcripts

Besides stabilizing the EBV life cycle and influencing host anti-EBV immune responses, EBV miRNAs also regulate growth and apoptosis of infected B cells (Table 1). To validate previously reported High-Throughput Sequencing of RNA isolated by Crosslinking Immunoprecipitation (HITS-CLIP) results, a screen was performed to identify EBV miRNAs that target Caspase 3 (CASP3) in Burkitt lymphoma cells. For eight out of twelve CASP3-targeting EBV miRNAs, direct interaction was confirmed using a luciferase reporter assay. For five of these miRNAs—BART22, BART1-3p, BART2, BART7, and BART8—a decrease in CASP3 protein was observed in HEK293T cells upon overexpression of the respective miRNA [50]. Targeting of CASP3 by BART16 was confirmed in another study [51]. Consistent with these findings, increased CASP3 protein expression was observed upon knockout of all BART miRNAs simultaneously [35].

In a functional screen focused on the identification of EBV miRNAs that block growth-promoting B-cell receptor (BCR) activation, six miRNAs (BHRF1-2, BART1, BART2, BART8, BART11, and BART18) were identified that inhibit the activity of the downstream NF-kB [52]. Additional testing of miR-BART9 and miR-BART17, selected based on previous studies, resulted in decreased NF-κB signaling upon anti-IgM stimulation, but not in the absence of stimulation. Based on previously published Photoactivatable Ribonucleoside-Enhanced Crosslinking and Immunoprecipitation (PAR-CLIP) data, multiple cellular targets were identified, including genes related to NF-kB signaling [52].

PTEN levels were elevated in EBV-Δ123 (BHRF1 miRNA KO) strain-infected cells compared to EBV wild-type-infected cells. Infection with the EBV-Δ123 strain led to increased apoptosis and reduced cell growth, especially during the initial weeks. The authors demonstrated that these BHRF1 miRNAs directly downregulate PTEN [44]. Knockdown of miR-BHRF1-2 in LCLs resulted in a G1 to S phase cell cycle arrest, likely due to the relief of PRDM1 repression, a transcriptional regulator involved in B-cell differentiation [53]. In addition, GRB2, a gene that regulates BCR signaling, was also identified as a target of miR-BHRF1-2-5p. Interestingly, the knockdown of GRB2 in LCLs lacking miR-BHRF1-2 partially restored the observed growth disadvantage [52].

**Table 1:** Overview of EBV miRNA-affected processes and reporter assay-confirmed EBV miRNA target gene interactions.

Process/ Target Gene	miRNA	Validated in	Ref.
Latency establishment	BHRF1 cluster	BL	[37]
Transition from latent to lytic infection	BART2, BART20-5p, BART Cluster 1	BL, Primary human B-cells	[35,40,41]
BCR signal transduction	BART1, BART2, BART8, BART9, BART11, BART17, BART18, BHRF1-2	BL, LCLs	[52]
BALF5 *	BART2	BL	[40]
BZLF1	BART20-5p	BL	[41]
BRLF1	BART20-5p	BL	[41]
Dicer1	BART6-5p	LCLs, BL	[42]
IL-12B IFI30 LGMN CTSB	BART1, BART2, BHRF1-2	B-lymphocytes	[46]
TAP2 *	BHRF1-3, BART17	Primary human B-cells	[47]
IL1R1 *	BHRF1-2-5p	LCLs, BL	[48]
PD-L1 *	BHRF1-2-5p	LCLs	[49]
PD-L2 *	BHRF1-2-5p	LCLs	[49]
CASP3	BART16, BART22, BART1-3p, BART2, BART7, BART8	LCLs	[50,51]
GRB2 *	BHRF1-2-5p	BL, LCLs	[52]
PRDM1 *	BHRF1-2-3p	LCLs	[53]
PTEN *	BHRF1 Cluster	LCLs, BL	[44]
LZTS2 *	BHRF1-1, BART2-5p	EBV + PTLD-related patient samples	[54]

* Indicates additional validation of miRNA target by Western blotting or other assays. BHRF1: BamHI fragment H rightward open reading frame 1; BART: BamHI-A rightward transcript; BL: Burkitt lymphoma; DLBCL: diffuse large B-cell lymphoma; PTLD: post-transplant lymphoproliferative disorder; LCLs: lymphoblastoid cell lines; Dicer1: Dicer ribonuclease 1 gene (gene symbol DICER1); IL-12B: interleukin-12 subunit beta; IFI30: interferon gamma inducible protein 30; LGMN: legumain; CTSB: cathepsin B; TAP2: transporter associated with antigen processing 2; IL1R1: interleukin 1 receptor type 1; PD-L1/PD-L2: programmed death-ligand 1/2; CASP3: caspase 3; BCR: B-cell receptor; GRB2: growth factor receptor-bound protein 2; PRDM1/Blimp1: PR domain containing 1; PTEN: phosphatase and tensin homolog; LZTS2: Leucine zipper tumor suppressor 2.

## 5. EBV miRNAs in B-Cell Lymphomas

The primary distinction in the classification of mature B-cell lymphoma is between Hodgkin lymphoma and all other lymphomas, collectively referred to as non-Hodgkin lymphoma. EBV is associated with Hodgkin lymphoma and some non-Hodgkin lymphoma entities, including Burkitt lymphoma, diffuse large B-cell lymphoma, and immunodeficiency-related lymphoproliferative disorders, such as post-transplant lymphoproliferative disorders (Table 2).

Hodgkin lymphoma is classified into classic Hodgkin lymphoma and nodular lymphocyte-predominant types. Tumor cells in classic Hodgkin lymphoma originate from germinal center B cells and are scarce (often less than 1% of the affected tissue) amid a large reactive infiltrate [8]. EBV infection is found in 30–40% of classic Hodgkin lymphoma cases in Western countries, but not in nodular lymphocyte-predominant Hodgkin lymphoma. Higher incidence of EBV has been reported in mixed cellularity subtype, elderly, and HIV-associated cases. EBV is thought to contribute to the early steps of lymphomagenesis by rescuing crippled GC-B cells from apoptosis [55,56]. The typical EBV latency pattern in classic Hodgkin lymphoma is type II, with expression of EBERs, EBNA1, LMP1, LMP2A, and LMP2B [10].

Burkitt lymphoma is an aggressive lymphoma originating from germinal center B cells and characterized by a chromosomal translocation of the MYC gene locus [57]. Endemic Burkitt lymphoma is highly prevalent in equatorial and sub-Saharan Africa and New Guinea, occurs at a median age of 6 years, and nearly all cases are EBV-positive [58]. Sporadic Burkitt lymphoma cases are observed in the USA and Western Europe with an overall incidence of three cases per million people per year. It has a bimodal age distribution, with peaks in pediatric and elderly populations [59]. The fraction of EBV-positivity in sporadic Burkitt lymphoma ranges from 10% to 30% [59]. Burkitt lymphoma can also arise in an immunodeficiency setting, most commonly in patients with HIV [60]. EBV typically exhibits a latency type I restricted expression program in Burkitt lymphoma, with expression restricted to EBNA1 [5].

Diffuse large B-cell lymphoma is a heterogeneous disease accounting for 30–40% of all newly diagnosed non-Hodgkin lymphomas [61]. Infection with EBV is found in a small subset (<10%) of diffuse large B-cell lymphoma cases, which are referred to as EBV-positive diffuse large B-cell lymphoma [62,63]. EBV-positive diffuse large B-cell lymphoma cases express all EBNA proteins (1, 2, 3A, 3B, 3C, and LP), three LMPs, and EBERs, consistent with a latency type III infection [64].

Post-transplant lymphoproliferative disorders are rare, heterogeneous lymphoid proliferations that develop in the setting of immunosuppressive therapy following solid organ transplantation or hematopoietic stem cell transplantation. The latest WHO classification places post-transplant lymphoproliferative disorders among lymphomas linked to immune deficiency and immune dysregulation [57]. About 50% of post-transplant lymphoproliferative disorder cases in solid organ transplantation recipients and 30% in hematopoietic stem cell transplantation are EBV-positive and develop as a result of viral reactivation or primary infection [57,65]. EBV-positive post-transplant lymphoproliferative disorders show latency type III, expressing EBERs and all EBNA and LMPs [8].

### 5.1. Profiling and Genomic Studies

Transcriptome and miRNA profiling studies indicated that the transcriptional landscape of EBV-positive Burkitt lymphoma is primarily influenced by EBV miRNAs. In contrast, the landscape of post-transplant lymphoproliferative disorders was largely defined by the expression of viral proteins [19]. The authors suggested different pathogenetic mechanisms of transcriptional regulation depending on the condition of the interactions between the host and EBV [19]. A follow-up study of 30 immunodeficiency-associated Burkitt lymphoma cases revealed distinct gene and miRNA expression profiles in EBV-positive compared to EBV-negative cases [66]. Mundo et al. reported expression of 19 EBV miRNAs in 4 EBV-positive Burkitt lymphoma samples but unexpectedly also observed low levels of EBV miRNAs in 6 EBV-negative cases. The authors suggested that the presence of EBV miRNAs indicated EBV infection in these Burkitt lymphoma cases, even though they were initially diagnosed as EBV-negative [67].

A large variety of genomic aberrations have been reported in the EBV genome, including both single-nucleotide variants and larger structural variants [34]. Analysis of EBV genomes isolated from various EBV-related diseases (*n* = 990) revealed a high prevalence of deletions in chronic active EBV disease, diffuse large B-cell lymphoma, extranodal natural killer/T-cell lymphoma, and Burkitt lymphoma (25–48%). In contrast, much lower frequencies were observed in patients with infectious mononucleosis, epithelial malignancies, and post-transplant lymphoproliferative disorders (around 5–11%) [68]. Deletions specifically involving the EBV miRNA clusters were less common and are described in more detail below.

### 5.2. EBV-miRNAs in Classic Hodgkin Lymphoma

Twelve BART miRNAs (with 10 being derived from cluster 2) were shown to be expressed in total tissue samples of 3 EBV-positive classic Hodgkin lymphoma, while being undetectable in EBV-infected normal GC and memory B cells [69]. In another study, profiling of total classic Hodgkin lymphoma tissue from two EBV-positive cases revealed expression of approximately half of the EBV miRNAs. Validation by RT-qPCR demonstrated the highest expression of miR-BART-13-3p in six classic Hodgkin lymphoma cases [70]. Nevertheless, there is no conclusive evidence that only a subset of BART miRNAs is expressed in classic Hodgkin lymphoma. These observations may reflect technical limitations in detecting EBV miRNAs, particularly given the low number of EBV-positive cells in the samples [71]. Two studies have reported deletions of BART miRNA clusters in classic Hodgkin lymphoma samples. In the first study, a 314 bp deletion in the EBV genome was described, encompassing part of the BART miRNAs [72]. In the second study, two out of 48 classic Hodgkin lymphoma samples harbored deletions in BART miRNA clusters, and one case showed a deletion in the BHRF1 miRNA region [68]. Although these remain rare observations, they highlight the need for further studies focusing on EBV-positive classic Hodgkin lymphoma.

### 5.3. EBV-miRNAs in Burkitt Lymphoma

Knockdown of miR-BART6-3p in EBV-positive Burkitt lymphoma resulted in reduced levels of PTEN and the p80 and gp130 chains of the IL-6 receptor (IL6R) [73]. In silico analyses suggested direct targeting of PTEN by miR-BART6-3p [66]. Genome-wide expression profiling after miR-BART6-3p knockdown in Burkitt lymphoma cells revealed multiple differentially expressed genes, including PTEN. PTEN was shown to be downregulated in primary EBV-positive Burkitt lymphoma cases compared to EBV-negative Burkitt lymphoma cases [74]. In another study, knockdown of miR-BART6-3p resulted in a decrease in Burkitt lymphoma cell growth and an increase in apoptotic cells [75]. In line with a potential regulatory role of miR-BART6-3p on IL6R, significantly lower levels of IL6R mRNA and protein were observed in EBV-positive compared to EBV-negative Burkitt lymphoma cases [75]. However, reporter assays to prove a direct interaction between miR-BART6-3p and the IL-6R chains were not conclusive [75].

Knockout of miR-BART7 and miR-BART9 using a CRISPR-Cas9 approach in Burkitt lymphoma cells resulted in a considerable decrease in cell viability and proliferation rate [76]. It remains to be determined whether these effects can be fully attributed to the knockdown of the miRNAs or if they are (partly) due to the induction of a DNA damage response caused by the cleavage of the estimated 20 copies of the EBV genome present in the cell line [77]. In addition to the reported effects on growth, increased levels of the viral transcripts for the lytic proteins BZLF1 and gp350 were observed compared to cells with normal levels of these two miRNAs [76]. Proteomics analysis following knockdown of miR-BART7-3p and miR-BART9-3p revealed 59 and 44 differentially expressed proteins, respectively, with each miRNA having distinct enriched pathways [76].

Two Burkitt lymphoma cell lines that express dominant negative EBNA1 (dnEBNA1) show a gradual loss of EBV episomes. Introducing BART1, BART3 through BART20, and BART22 miRNAs in these cells enhanced proliferation compared to control cells. Ectopic expression of miR-BART-1 and miR-BART-16 reduced global cell death and blocked apoptosis. This effect was associated with lower levels of CASP3 [51], a direct target of both miR-BART1-3p [50] and BART-16-5p [51]. Together, these results show that EBV miRNAs, particularly BARTs, can sustain Burkitt lymphoma growth.

### 5.4. EBV-miRNAs in Diffuse Large B-Cell Lymphoma

Profiling of EBV miRNAs in HIV-associated EBV-positive diffuse large B-cell lymphoma revealed high expression for miR-BART2 [78]. In another study, it was shown that EBV-positive diffuse large B-cell lymphoma expressed most miRNAs from the BART cluster, except for miR-BART15 and miR-BART20 [79]. In contrast to the EBV miRNA expression patterns observed in latency type III-infected cells, no expression was observed for the miR-BHRF1 cluster in EBV-positive diffuse large B-cell lymphoma [79]. This might be attributed to the technical limitations of the miRNA cloning technique used for quantification in this first profiling study [79]. In contrast, the levels of BHRF1-3 were high in HIV-associated EBV-positive diffuse large B-cell lymphoma [70,78] and inversely correlated with IFN-inducible T-cell-attracting chemokine CXCL-11/I-TAC levels [78]. Suppression of CXCL-11 expression was reversed by inhibiting miR-BHRF1-3, while CXCL-11 mRNA and protein levels were reduced upon overexpression of BHRF1-3. This indicated potential targeting of CXCL-11 by miR-BHRF1-3. However, the results of reporter assays did not support direct interaction [78]. Although not definitive, these results highlight a potential link between miR-BHRF1-3 expression and immune modulation in EBV-positive diffuse large B-cell lymphoma.

Deletions specifically involving the BART miRNA clusters were reported in about 30% of EBV-positive diffuse large B-cell lymphoma cases, while the percentages were much lower in Burkitt lymphoma, classic Hodgkin lymphoma, and post-transplant lymphoproliferative disorders (around 1–3%). Partial deletions of BART miRNA cluster 1 were most common and included miR-BART6, a miRNA known to control lytic activation [68]. Two other EBV miRNAs often included in the deleted region, i.e., miR-BART-20 and miR-BART-2, are also implicated in the control of the lytic activation (see Section 4.1). Interestingly, the observations in EBV-positive diffuse large B cell lymphoma resemble the deletions observed in the B95.8 EBV strain, which also includes miR-BART6 and miR-BART-20 [68]. Altogether, these findings suggest that genomic deletions of EBV miRNA regions are specifically linked to diffuse large B-cell lymphoma and seem to be less relevant for the pathogenesis of other EBV-associated B-cell lymphomas.

### 5.5. EBV-miRNAs in Post-Transplant Lymphoproliferative Disorders

Profiling studies in primary central nervous system post-transplant lymphoproliferative disorder and systemic post-transplant lymphoproliferative disorder revealed expression of thirty-nine of the 44 EBV-miRNAs, consistent with the latency III program [80]. High levels of miR-BHRF1-1 and miR-BART2-5p were observed in EBV+ post-transplant lymphoproliferative disorder. These two miRNAs directly targeted leucine zipper tumor suppressor 2 (LZTS2) and thereby activated the PI3K-AKT pathway (Table 1) [54]. In seven primary post-transplant lymphoproliferative disorder cases, miR-BHRF1-2 and PRDM1 mRNA levels showed a positive correlation. In contrast, high miR-BHRF1-2 levels were associated with low PRDM1 protein levels, suggesting a post-transcriptional repression by miR-BHRF1-2 [53]. The authors indicated the potential relevance of their findings for EBV-positive diffuse large B-cell lymphoma, also showing decreased levels of PRDM1 protein [53]. These findings suggest that miR-BHRF1-2 contributes to post-transplant lymphoproliferative disorder pathogenesis by downregulating PRDM1 protein.

## 6. Integrated Regulatory Axes of EBV-miRNAs in B Cells

**Figure 2:**
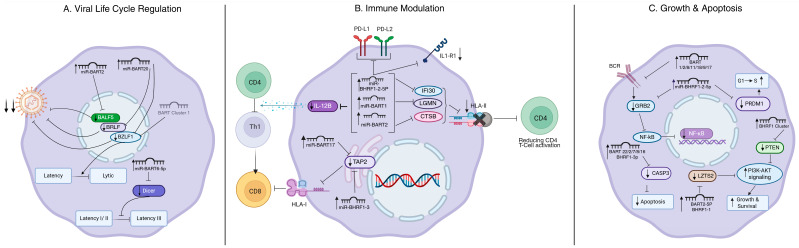
Schematic representation of the regulatory axes of EBV-encoded miRNAs in infected B cells. (**A**) Control of the viral life cycle by miRNA-mediated modulation of the latency–lytic balance and early transformation-associated transcripts. (**B**) Control of immune evasion by EBV miRNAs via repression of cytokine production, antigen processing and presentation, and modulation of CD4^+^ and CD8^+^ T-cell responses. (**C**) Control of growth and apoptosis by EBV miRNAs targeting mediators of apoptosis, components of B-cell receptor (BCR) signaling, PI3K–AKT signaling, and regulators of the cell cycle. Only experimentally validated, high-confidence targets are shown. Arrowheads indicate activation or signaling flow, whereas blocking lines indicate repression. The figure was created using BioRender.com.

Collectively, the available data support a critical role of EBV-encoded miRNAs in B cells and EBV-positive B-cell lymphoma (Figure 2). The three main regulatory axes, i.e., control of the viral life cycle, modulation of EBV-directed immune responses, and regulation of host cell growth and apoptosis, are all critically linked to specific EBV miRNAs or EBV miRNA clusters. MiR-BART1, miR-BART2, and miR-BHRF1-2 emerge as shared regulators that converge on central signaling hubs such as NF-κB, PI3K–AKT, and antigen presentation pathways (TAP2/HLA-II). These interactions are supported by direct experimental validation in multiple systems and illustrate functional redundancy and pathway convergence (Table 1).

Lymphoma subtype-specific functional roles of EBV miRNAs include BART/BHRF1-mediated apoptosis and PTEN regulation in Burkitt lymphoma, and BHRF1-driven PI3K-AKT activation and stronger immune modulation in EBV-positive diffuse large B-cell lymphoma/post-transplant lymphoproliferative disorder. For classic Hodgkin lymphoma, mechanistic validations are limited.

Since nasopharyngeal carcinoma shows abundant expression of BART miRNAs as well, it is of interest to contrast this disease to EBV-positive lymphomas, even though the cellular context is different. Some of the BART miRNAs likely have similar roles in both types of EBV-driven malignancies. This is, for instance, the case for viral targets of the BART miRNAs such as BALF5, BZLF1, and BRLF1 [40,41]. For other miRNAs, different roles have been described in B cells or B-cell lymphoma versus nasopharyngeal carcinoma. For example, miR-BARTs have been implicated in epithelial to mesenchymal transition, a process that has relevance to nasopharyngeal carcinoma only [81]. MiR-BART2 has been implicated to control antiviral CD4+ T cell responses via multiple targets in B cells [46]. In contrast, it was reported to promote metastasis by suppressing RND3 [82]. MiR-BART2 has been reported to repress Dicer1 in nasopharyngeal carcinoma [83], while Dicer1 is targeted by miR-BART-6-5p in B cells [42]. It remains to be determined to what extent these and other miRNA-target gene interactions are specific for B cells or epithelial cells and which ones are shared. A broader overview of EBV miRNA targets in nasopharyngeal carcinoma is given elsewhere [81]. Remarkably, nasopharyngeal carcinoma usually does not harbor EBV miRNA deletions, suggesting that it depends on intact BART clusters for epithelial persistence and immune modulation, while such deletions may favor the development of certain subtypes of B-cell lymphoma [68].

## 7. Challenges and Future Perspectives

In addition to the viral proteins essential for maintaining its life cycle and persistence, EBV encodes 44 miRNAs that target both viral and host genes. These EBV miRNAs contribute to viral persistence, immune evasion, and the regulation of oncogenic processes. This review summarizes the current understanding of EBV miRNA expression and function in B-cell lymphomas and their normal counterparts.

Expression profiling studies using primary EBV+ lymphoma cases have provided comprehensive transcriptional landscapes that were predominantly influenced by either EBV miRNAs or viral proteins [19]. To further confirm these specific patterns, additional studies including larger numbers of all EBV-positive B-cell lymphoma entities are necessary. It remains challenging to directly compare profiles of different lymphoma entities using tissue samples with each other and control tissues due to variations in the percentage of EBV+ (tumor) cells and variability in levels of cellular miRNAs. Profiling bulk-purified tumor cells or single cells might partly overcome these limitations.

Current knowledge on the role of EBV miRNAs in B-cell lymphoma is limited, as most studies have relied on LCLs rather than lymphoma cell lines or primary tumor samples. Nevertheless, several functions relevant to lymphoma biology have been identified (Table 1). Genome-wide miRNA-targetome identification studies combined with bioinformatics approaches have revealed numerous potential cellular targets of EBV miRNAs in Burkitt lymphoma [24], diffuse large B-cell lymphoma [84] and LCL models [25,85,86]. Notably, many EBV miRNAs share seed sequences with human miRNAs, such as those from the miR-17~92 cluster and miR-155 [24,27], enabling them to co-target or hijack key host gene regulatory networks. There is a substantial overlap in the cellular targets regulated by different EBV miRNAs, often converging on critical pathways regulating cell growth, apoptosis, the immune response, and, notably, the NF-κB signaling pathway. Additionally, EBV miRNAs contribute to lymphomagenesis by controlling the EBV life cycle and facilitating immune escape. However, Argonaute-crosslinking and immunoprecipitation (AGO-CLIP)–based miRNA-targetome data are currently lacking for EBV-positive classic Hodgkin lymphoma and post-transplant lymphoproliferative disorder. Although many putative miRNA–target interactions have been identified, only a small subset has been experimentally validated using luciferase reporter assays or Western blotting, mostly in LCLs. Future studies are required to confirm these interactions in lymphoma cell lines or primary lymphoma, coupling target validation with phenotypic rescue assays to dissect the relevance of these targets to lymphoma biology.

Understanding EBV miRNAs through functional analysis remains challenging due to the complexity and redundancy of the miRNA repertoire. Deletion strains, such as miRNA-null EBV or cluster-specific knockouts (BHRF1 and BART), have offered valuable insights [35,37]. However, these broad deletions may hide the roles of individual miRNAs. Knockout of individual miRNAs in EBV-positive cell lines is challenging due to the high number of EBV episomes. Blocking their function with antisense oligos seems more feasible but might not be effective enough. To truly dissect the specific roles of individual EBV miRNAs, the emphasis should be on generating a comprehensive library of EBV strains, each with a single miRNA knock-out. Such experimental model systems will enable precise analysis of the roles of individual miRNAs in B-cell infection, regulation of the EBV life cycle, regulation of the immune response, and lymphomagenesis.

Although important functions of some EBV miRNAs have been reported, especially in normal B-cells, the picture is far from complete. For example, the functionally relevant EBV miRNA targets in normal B cells often remain unknown. The contribution of EBV miRNAs to lymphoma development should be a particular focus of future research to enhance our understanding of how EBV miRNAs influence B-cell transformation and contribute to lymphoma biology.

## 8. Conclusions

The studies summarized in this review illustrate how EBV-encoded miRNAs contribute to the regulation of key processes that sustain EBV infection. Through coordinated targeting of both viral and host transcripts, these miRNAs participate in the control of viral persistence, modulation of immune responses, and regulation of cellular growth and survival pathways. For B-cell lymphoma clear links have been identified between EBV miRNAs and control of EBV latency patterns, immune escape, and support of growth. Overall, mechanistic support for the role of EBV miRNAs in EBV-associated lymphomas remains limited. Expanding functional studies in these disease contexts will therefore be important to clarify how EBV miRNAs contribute to lymphoma development across different EBV-associated malignancies.

## Figures and Tables

**Table 2:** Characteristics of EBV-associated B cell lymphomas.

Disease	EBV Association	Latency Type	EBV Profile				
			miR-BARTs	miR-BHRFs	EBNA	LMPs	EBER
Burkitt lymphoma	>95% * 10–30% **	I	+	−	EBNA1	−	+
Classic Hodgkin lymphoma	30–40%	II	+	−	EBNA1	All	+
Diffuse large B-cell lymphoma	<10%	III	+	+	All	All	+
Post-transplant lymphoproliferative disorder							
HSCT-associated	<30%	III	+	+	All	All	+
SOT-associated	50–80%						

+: expressed; −: not expressed; BamHI-A rightward transcripts (BART) microRNAs; BamHI fragment H rightward open reading frame 1 (BHRF1) microRNAs; EBNA: EBV nuclear antigens; LMPs: latent membrane proteins; EBER: EBV-encoded small RNAs; HSCT: hematopoietic stem cell transplantation; SOT: solid organ transplantation. * Formerly known as endemic BL, it is highly prevalent in equatorial Africa and New Guinea. ** Previously known as sporadic cases, it is observed in the USA and Western Europe.

## Data Availability

No new data were created or analyzed in this study.

## Supplementary Materials

The following supporting information can be downloaded at: https://www.mdpi.com/article/10.3390/cancers18060962/s1, Table S1: EBV miRNAs that share their seed sequences with human miRNAs [21,22,24,87].
