# Supplementary material for: Relevance of Epstein-Barr Virus (EBV) miRNAs in EBV-Infected B Cells and B-Cell Lymphomas
Source: Cancers (Basel). 2026 Mar 16;18(6):962. doi: 10.3390/cancers18060962 (PMC13025152; doi:10.3390/cancers18060962)
Supplement: Supplementary file 1 [file cancers-18-00962-s001.zip › cancers-4138481-supplementary.pdf]

cancers

MDPI

Supplementary Materials: Relevance of Epstein - Barr virus (EBV) miRNAs in EBV-Infected B Cells and B-Cell Lymphomas

Nohora Juliana Rueda-Forero, Joost Kluiver, Marije Koning, Anke van den Berg and Arjan Diepstra

Table S1. EBV miRNAs that share their seed sequences with human miRNAs.

EBV miRNA	Seed Sequence*	Human miRNAs	Reference
BART1-3p	AGCACCG	miR-29a/b/c	[22,24]
BART2-3p	AGGAGCG	miR-28-5p<br>miR-708	[22]
BART3-3p	GCACCAC	miR-767-5p	[22]
BART4-3p	ACAUCAC	miR-499-3p	[22]
BART5-5p	AAGGUGA	miR-18a/b	[21,22,24]
BART7-3p	AUCAUAG	miR-154-3p<br>miR-487a-3p	[22]
BART7-5p	CUGGACC	miR-378<br>miR-422a	[22]
BART8-3p	UCACAAU	miR-513b	[22]
BART9-3p	AACACUU	miR-141<br>miR-200a	[22,87]
BART9-5p	ACUGGAC	miR-1243	[22]
BART11-5p	CAGACAG	miR-1324	[22]
BART12-3p	CCUGUGG	miR-1914	[22]
BART22-3p	ACGGCUA	miR-520d-5p<br>miR-524-5p	[22,24]
BHRF1-1	AACCUGA	miR-490-3p<br>miR-649	[22]

*BART: BamHI-A rightward transcript microRNAs; BHRF1: BamHI fragment H rightward open reading frame 1 microRNAs. *Seed sequence: nucleotides 2-8 of mature miRNA.

 https://doi.org/10.3390/18060962
